# Dr. Walker's Observations on the Matter of Vacciola

**Published:** 1804-09-01

**Authors:** John Walker

**Affiliations:** Salisbury Square


### *To the Editors of the Medical and Physical Journal.*

GENTLEMEN,

LET me beg of you again to permit me to exercise a little of what some may be ready to term hyper-criticism. Your candour will more readily incline you to yield to my earnest request when you find that my observations are no longer upon places where, or persons who, but directly *ad rem,* to the thing in which the inhabitants of the whole world are interested.

In page 182, (Aug. 1804,) ye say, "Allowing the facts, the contradiction can only be explained by admitting the possibility of both perfect and imperfect vaccination, from a vitiated source, under similar circumstances, and in each case with an entirely regular progress of the inoculated vesicle."

How will this be understood by your readers in general? Are we *ipso facto* to admit such possibility? or are we only to admit it *ad absurdum* or *ex absurdo*? From more vexatious and teazing experience than I hope has fallen to the lot of any other individual, I have learnt that perfect cow-pock cannot possibly be produced from a vitiated source; while it is a fact that matter taken from the centre of a perfect pock, and of a proper age, may utterly fail to produce the desired effect, though most dexterously applied with the lancet.

Every body is acquainted with the efforts of nature in the reparation of injuries; the formation of callus after fractures, of cicatrix after the destruction of soft parts. I believe the epidermis is not united after being cut, abraded, or lacerated, without its formation of cicatricle. It is this perhaps which is principally the occasion of the characteristic depression in the centre of the cow-pock, from its earliest formation. In the incipient pock the cicatricle

 does does not allow it to swell out full on its summit. When it has acquired its full dimensions it might be expected to be flat, like extensive blisters from a scald, while smaller ones are nearly spherical. At such time we know the variolous pock breaks into a number of distinct vesicles, while the vacciolous one,* (or rather the vacciolous congeries of cells) which it is necessary to break up by various punctures in obtaining the guardian fluid, keeps united. If the inoculation have been neatly performed with a sharp instrument, the genuine vacciolous fluid is immediately obtained on puncturing it with the lancet, and can never be taken too soon. If by many punctures and pressures on the pock an increased flow of fluid be promoted, the lymphatic discharge so excited may afford an ichor or virus somewhat diluted; and if by the wounding of some ramification of blood-vessel it become distained still further dilution is occasioned; but such matter, in a thousand instances, I have found to produce only the genuine effect. On the other hand, if the inoculation have been performed by an untoward application of the instrument, or by too heavy a hand, an action is set up which gives a different appearance to the pock. A small scab is soon formed at the place

---

* Why should we say Vaccine, &c. *a la Francoise?* The world is much indebted to the French chemists for their new nomenclature; but, as we lay aside our term cow-pock, of Gothic origin, and adopt a technical (classic) one; either let us follow the original one of Jenner, variola vaccina, or the more happy one of Stokes, vacciola. From the villages all about town we have milk; from Cambridgeshire, butter; from Cheshire and Gloucestershire, cheese. All this is vaccine matter; and, *a fortiori*, the flesh of the animal is so; but vacciolous matter is matter of vacciola or cow-pock, which rather better admits of all the changes of verb, participle, adjective, &c. being rung upon it than *vaccin*. I hope ye will more steadily adhere to the use of it, in opposition to the French inaccuracy, which many of our most eminent vacciolators so complaisantly adopt; (thus shewing that the greatest physiological skill by no means implies or promises philological acumen) I hope this, because I consider your Journal as the most extensively spread medical work we have. At any rate it will be well for you, when ye make mention of the important subject, yourselves, to adhere steadily to one or other of the two terms; otherwise all your movements will appear extremely *vaccillatory*, which would be but very badly suited to your own *unwavering* conviction on the efficacy of vacciolation.

From Ring's elaborate "Treatise on the Cow-pox," I extract the French Nomenclature, which I am sorry to have seen him follow in so valuable a work, in order to contrast it with the more happy one which naturally arises from the root Vacciola of Stokes.

| French. | Stokes's. | English. |
|---|---|---|
| *Vaccine.* | *Vacciola.* | Cow-pock. |
| *Vaccin.* | Vacciolous matter. | Cow-pock matter. |
| *Vaccination.* | Vacciolation. | Cow-pock inoculation. |
| *Vacciner.* | To vacciolate. | To inoculate cow-pock. |

place of inoculation, underneath which a quantity of pus is collected in the cavity, which it forms and fills, in the centre of the pock. If the lancet be charged with this matter only, no effect is produced ; and in this way we must account for those failures which sometimes happen in inoculating from an otherwise perfect vesicle; while matter taken from punctures made in the sides of such pock produces the perfect effect. If the scab be removed from the pock, and the pus be wiped away, it then represents in form, if we may be allowed *magnis componere parva*, the crater of a volcano, rather than any thing like a regular tumulus; and on being broken down with the lancet, gives, in diminished quantity, a fluid equally active with that from the uninterrupted and fully formed pock.

Respectfully,

JOHN WALKER.

*Salisbury Square*, 12, *viij*. 1804.

